# Cytosolic serine hydroxymethyltransferase controls lung adenocarcinoma cells migratory ability by modulating AMP kinase activity

**DOI:** 10.1038/s41419-020-03215-0

**Published:** 2020-11-26

**Authors:** Amani Bouzidi, Maria Chiara Magnifico, Alessandro Paiardini, Alberto Macone, Giovanna Boumis, Giorgio Giardina, Serena Rinaldo, Francesca Romana Liberati, Clotilde Lauro, Cristina Limatola, Chiara Lanzillotta, Antonella Tramutola, Marzia Perluigi, Gianluca Sgarbi, Giancarlo Solaini, Alessandra Baracca, Alessio Paone, Francesca Cutruzzolà

**Affiliations:** 1 Department of Biochemical Sciences A. Rossi Fanelli, Laboratory Affiliated to Istituto Pasteur Italia, Sapienza University of Rome, Piazzale A. Moro 5, 00185 Rome, Italy; 2 Department of Physiology and Pharmacology V. Erspamer, Sapienza University of Rome, Piazzale A. Moro 5, 00185 Rome, Italy; 3 Department of Biomedical and Neuromotor Sciences, University of Bologna, Via Irnerio 48, 40126 Bologna, Italy; 4 Present Address: Department of Biosciences, Biotechnologies and Biopharmaceutics, University of Bari “Aldo Moro”, Via Orabona 4, 70121 Bari, Italy

**Keywords:** Cancer metabolism, Cell migration

## Abstract

Nutrient utilization and reshaping of metabolism in cancer cells is a well-known driver of malignant transformation. Less clear is the influence of the local microenvironment on metastasis formation and choice of the final organ to invade. Here we show that the level of the amino acid serine in the cytosol affects the migratory properties of lung adenocarcinoma (LUAD) cells. Inhibition of serine or glycine uptake from the extracellular milieu, as well as knockdown of the cytosolic one-carbon metabolism enzyme serine hydroxymethyltransferase (SHMT1), abolishes migration. Using rescue experiments with a brain extracellular extract, and direct measurements, we demonstrate that cytosolic serine starvation controls cell movement by increasing reactive oxygen species formation and decreasing ATP levels, thereby promoting activation of the AMP sensor kinase (AMPK) by phosphorylation. Activation of AMPK induces remodeling of the cytoskeleton and finally controls cell motility. These results highlight that cytosolic serine metabolism plays a key role in controlling motility, suggesting that cells are able to dynamically exploit the compartmentalization of this metabolism to adapt their metabolic needs to different cell functions (movement vs. proliferation). We propose a model to explain the relevance of serine/glycine metabolism in the preferential colonization of the brain by LUAD cells and suggest that the inhibition of serine/glycine uptake and/or cytosolic SHMT1 might represent a successful strategy to limit the formation of brain metastasis from primary tumors, a major cause of death in these patients.

## Abstract (web summary)

Lung cancer is a very aggressive tumor that often forms brain metastases. We show that lung cancer cells motility, fundamental for the formation of metastases, is controlled by amino acids such as serine and glycine, abundant in brain microenvironment.

## Introduction

Metastasis is the major factor responsible for the death of cancer patients. Lung cancer is a highly aggressive tumor and clinical evidence demonstrates that it successfully metastasizes to brain, liver, bones, and adrenal glands^1^. Approximately 30% of patients with lung adenocarcinoma (LUAD) show brain metastases (BM-LUAD) already at the time of diagnosis and 50% of them will eventually develop brain metastases. Treatment options for BM-LUAD are few and of limited efficacy, suggesting that more focused efforts are needed to study the molecular aspects of brain colonization by metastatic cells and to identify suitable therapeutic targets.

Metastasizing cells undergo metabolic rewiring when they enter and colonize a distal organ, either to support increased demand for ATP and biomass for proliferation, or as a result of differential nutrient and oxygen availability. Given that this metabolic shift is instructed by the colonized organ, it is crucial to understand which metabolic vulnerabilities cancer cells develop during metastasis, and which might differ from those observed in the tissue of origin. As seen for other tissues, also brain metastases show metabolic flexibility, mimicking the neuronal metabolic profile^2^. They adapt by using different metabolic strategies, such as upregulation of gluconeogenesis enzymes in response to lower glucose availability, utilization of glutamine and branched chain amino acids for ATP and biomass production^3^, and utilization of the pentose phosphate pathway or GABA metabolism to support nucleotide biosynthesis and the Krebs cycle^4,5^.

The metabolic pathways involving the nonessential amino acids serine and glycine are known to control the proliferation of cancer cells^6–9^. Import of extracellular serine guarantees the high proliferation rates of many tumors^10,11^. Serine starvation impairs cell viability due to unbalanced redox defense^12^, a feature which, alone or in combination with other strategies, can be exploited to control cancer growth^13,14^. Little is known about how serine and glycine produced in a specific microenvironment may affect the choice of that organ for the development of metastases.

In this paper, we have studied if and how the presence of serine and glycine in the extracellular milieu is able to affect the motility of LUAD cell lines. Utilizing a brain-derived extracellular fluid, we show that these metabolites, present in this milieu, are able to drive cell motility, and that inhibitors of the corresponding transporters can block the process. Our results also prove that the cytosolic isoform of the one-carbon metabolism (OCM) enzyme serine hydroxymethyltransferase (SHMT) controls this process by modulating cytosolic serine levels, which, in turn, control AMP kinase and the cytoskeleton dynamic processes, necessary for cell motility. Based on our observations, we propose a working hypothesis on how these metabolites may control the migration of lung cancer cells into the brain.

## Results

### Serine and glycine intracellular levels affect the migratory properties of lung adenocarcinoma cells

**Fig. 1:**
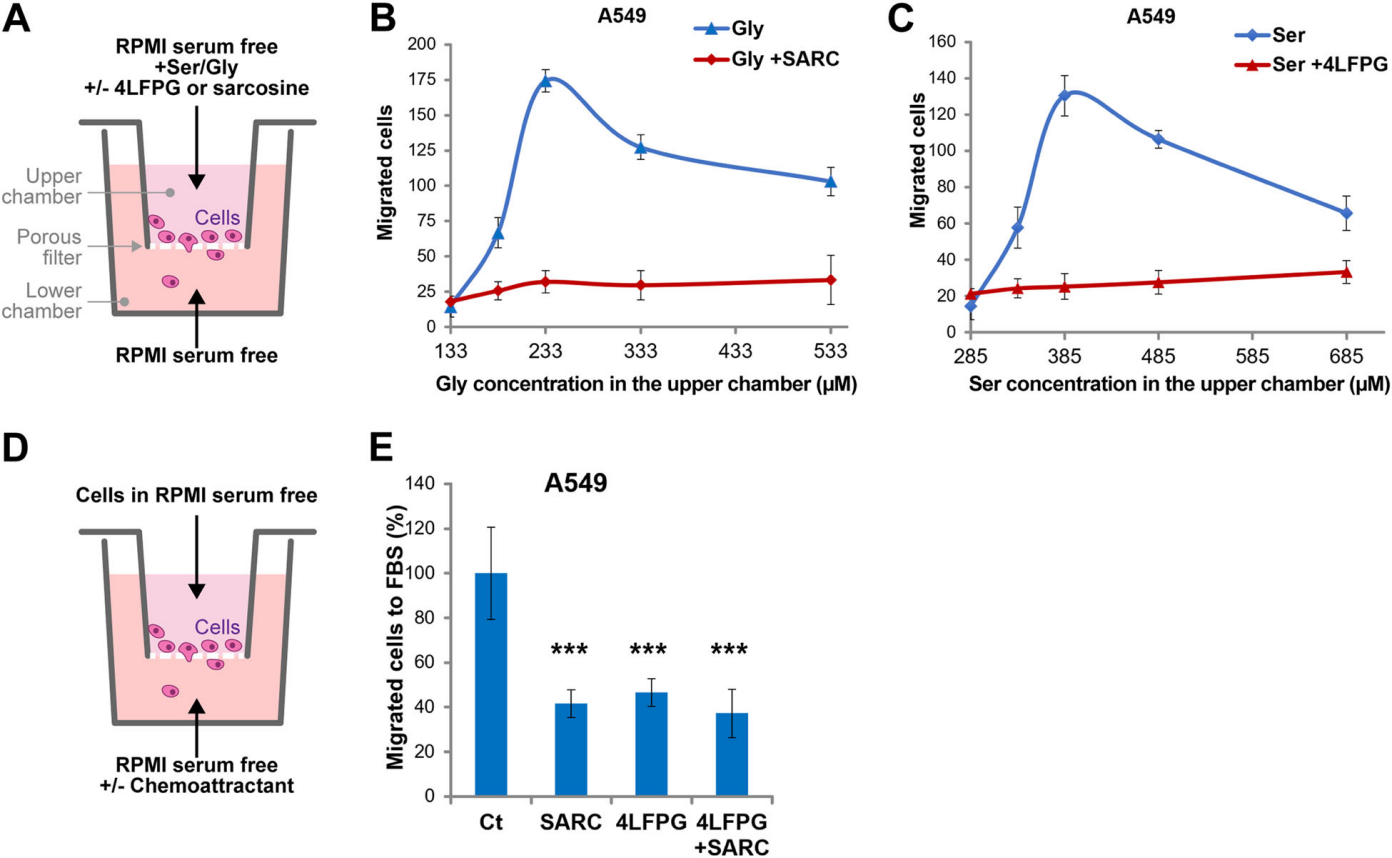
Serine and glycine intracellular levels affect the migratory properties of lung adenocarcinoma cells. **A** Scheme of competitive cell migration test using the Boyden chamber. The cells were resuspended in RPMI serum free (that initially contains 285 μM serine (Ser) and 133 μM glycine (Gly)) and seeded in the upper chamber, supplemented with increasing concentrations of Ser or Gly (0, 50, 100, 200, and 400 μM) in presence or not of 25 μM of 4LFPG or SARC, respectively. The migrated cells were then counted and represented as a function of amino acid concentration in (**B**) for Gly and in (**C**) for Ser (*n* = 3 each). **D** Schematic representation of chemoattraction bioassay test using Boyden chamber. The cells were placed in RPMI serum free in the upper chamber, while the lower chamber was filled with RPMI serum free plus chemoattractant 50% FBS or 100% BEF. Migration test of A549 cells to 50% FBS, pretreated or not for 3 h with SARC and/or 4LFPG. The migrated cells were then counted and represented in (**E**) (*n* = 4).

The influence of serine and glycine present in the culture medium Roswell Park Memorial Institute (RPMI) on the chemokinesis of LUAD A549 cells was evaluated using Boyden chamber assay (see scheme in Fig. 1A). The intracellular level of these amino acids was varied by increasing their concentration in the medium or by reducing their uptake by employing 4-Fluorophenyl-L-glycine (4LFPG) and sarcosine (SARC), which inhibit serine and glycine importers SLC1A4/SLC1A5 and GlyT1, respectively^15,16^. Using these inhibitors, we observed a reduction of the intracellular content of serine and glycine of about 18% for 4LFPG and 40% for SARC (Supplementary Fig. 1A). The increase in serine and glycine increases the migration of cancer cells in the absence of a chemoattractant, the maximum effect being observed in the presence of 233 μM glycine and 385 μM serine (Fig. 1B, C, blue lines). On the contrary, inhibition of the transporters blocks the migratory process (Fig. 1B, C, red lines) without significantly altering cell survival even after 72 h of treatment (Supplementary Fig. 1B). These experiments clearly indicate that the presence of these amino acids in the environment influences the ability of A549 cells to move.

To further study the effect of extracellular serine and glycine on the chemoattractant-induced migration process, we used fetal bovine serum (FBS), known to be a powerful chemoattractant for cancer cells (Fig. 1D)^17^. While in the presence of 50% FBS the A549 cells are strongly attracted, addition of inhibitors of serine and glycine uptake dramatically reduces the migratory ability of the cells, showing that the selected inhibitors are effective also in presence of FBS as an attractant (Fig. 1E). Other adenocarcinoma cell lines such as H1299 and H460 cells display a similar behavior (Supplementary Fig. 1C).

### Serine and glycine control the migration process induced by the brain extracellular fluid (BEF)

**Fig. 2:**
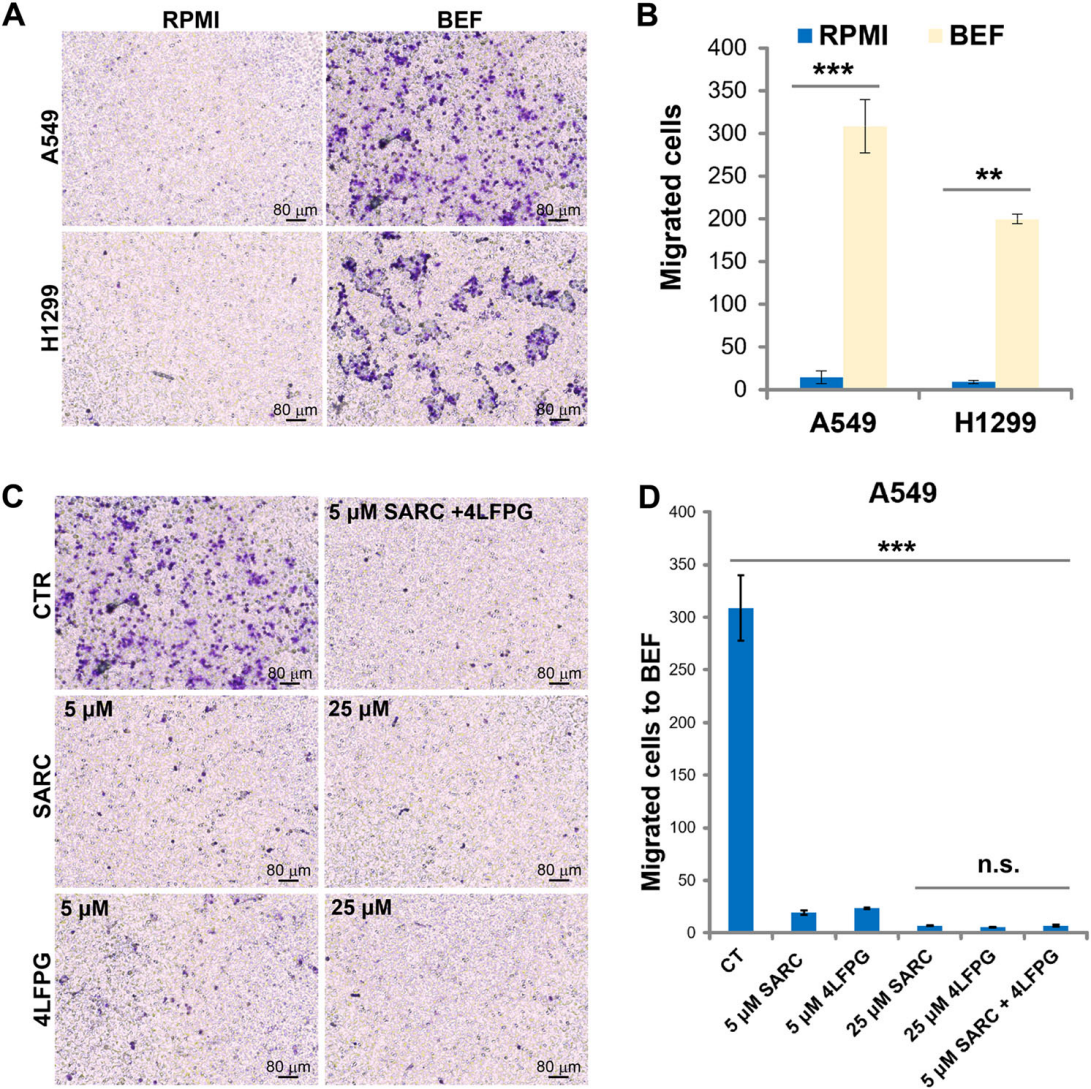
Serine and glycine control the migration process induced by the BEF. The A549 and H1299 cells migrated to BEF (images in (**A**)) were counted and the results plotted in comparison with non-supplemented RPMI (**B**) (*n* = 3 for both cell lines). Images of A549 cells pretreated or not for 24 h with SARC and/or 4LFPG are shown in (**C**) and counted in (**D**). Data are mean ± SD (standard deviation) of three separate sets of biological experimental replicates. **P < 0.01; ***P < 0.005.

The brain is, together with the bone, liver, and the adrenal glands, the organ of choice for the establishment of metastases that originate from pulmonary adenocarcinoma^1^. To study the involvement of serine and glycine in the migration process to the brain, we used a dissection protocol of mouse brain tissue commonly used to prepare brain primary cells, followed by disposal of the solid part and collection of the extracellular fluid (hereinafter BEF). Pulmonary adenocarcinoma cells A549 and H1299 are strongly attracted to BEF with respect to non-supplemented RPMI (Fig. 2A, B). The metabolite composition of BEF was determined using gas mass spectrometry (gas-MS) (Supplementary Table 1); the results show that BEF is enriched in metabolites, such as lactate, typically secreted by brain cells^18^. The level of several amino acids in BEF remains below the detection limit of the MS instrument, suggesting that only a limited number of cells are broken during the preparation. Considering the striking chemotactic effect of BEF on lung cancer cells and the importance of serine and glycine in the observed migratory process, we evaluated the concentration of these amino acids in BEF, by employing calibration curves. In the different preparations of BEF, the concentration of serine and glycine was found to be 42.5 ± 28.79 μM and 51 ± 14.91 μM, respectively, in agreement with the concentration previously reported for the cerebrospinal fluid^19^. As shown for FBS, the addition of 4LFPG and SARC dramatically reduces the migratory ability induced by BEF, demonstrating that the presence of these amino acids in the extracellular environment is required for the movement of LUAD cells (Fig. 2C, D).

### SHMT1 controls serine/glycine cytosolic interconversion and affects cell migration

To understand the link between the observed increase in cell migratory ability and the levels of serine and glycine, we further investigated their intracellular metabolism. Serine and glycine are involved in the OCM pathway, including key metabolic routes that provide nucleobases, antioxidant power, and methyl donors^20^. Serine and glycine are interconverted by the enzyme SHMT, which maintains the correct distribution of these metabolites in the cytosol and mitochondria, thanks to two SHMT isoforms located in these compartments and named SHMT1 and SHMT2, respectively. To clarify the details of serine/glycine compartmentalized interconversion and its involvement in the migratory process, we performed knockdown experiments by RNAi on both SHMT isoforms in A549 cells (Supplementary Fig. 2A)^21^ and then assayed the migratory ability as described above.

**Fig. 3:**
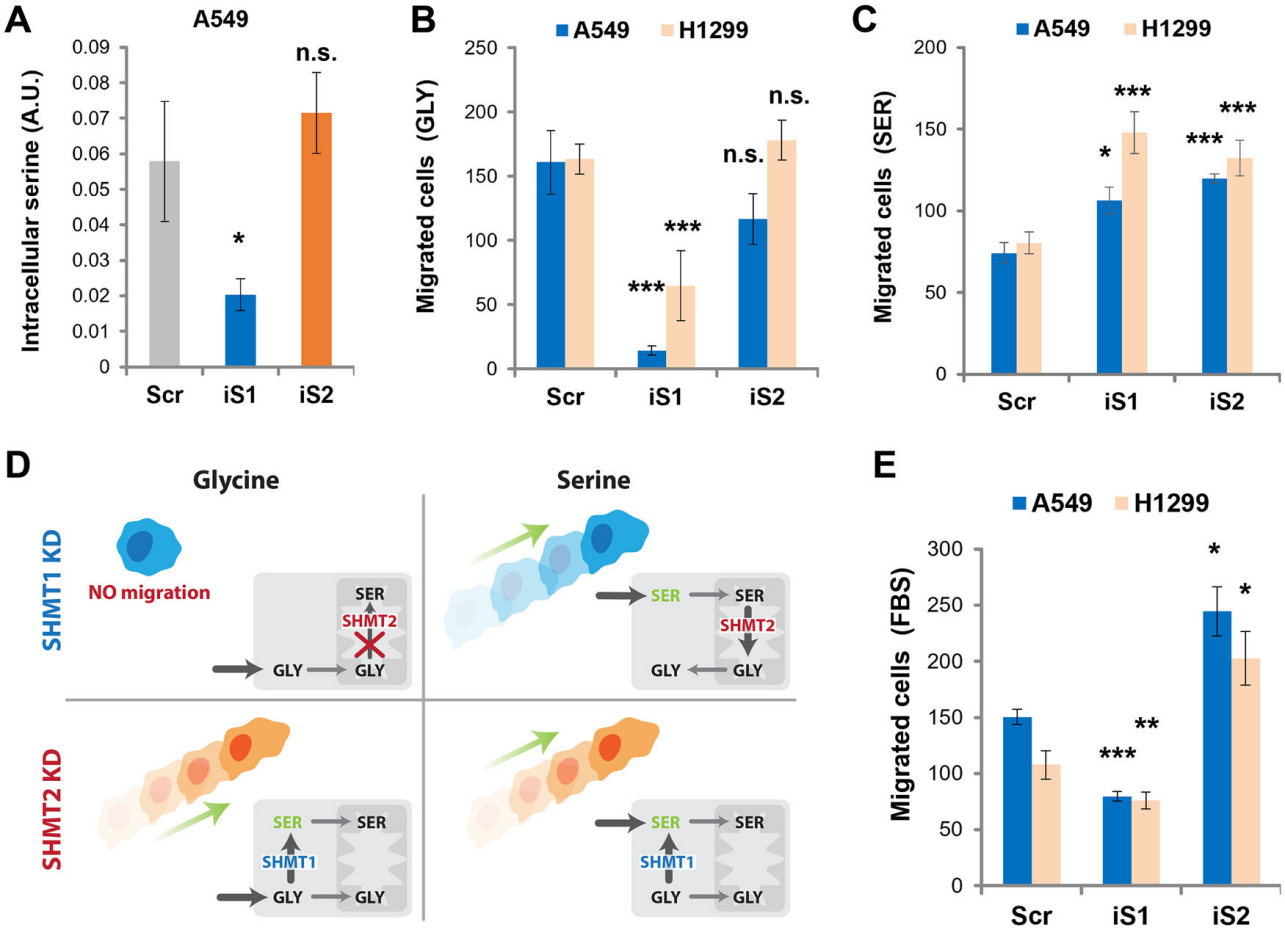
SHMT1 controls glycine-to-serine cytosolic interconversion and affects cell migration. **A** Intracellular serine produced in A549 cells transfected with siRNA against either SHMT1 (iS1) or SHMT2 (iS2) measured by gas-MS, as compared to cells transfected with a scramble sequence control (Scr) and grown for 48 h in MEM supplemented with 400 μM Gly and 500 μM Formate (*n* = 3). Migration test toward Gly (**B**), Ser (**C**), or FBS (**E**) was carried out with A549 cells transfected as indicated. The graphs represent three experimental replicates (*n* = 3 for all the experiments). **D** Scheme summarizing the results obtained by knocking down SHMT1 (SHMT1 KD) or SHMT2 (SHMT2 KD) on cytosolic Ser levels and cell motility.

Both isozymes are able to catalyze the reversible conversion of serine to glycine^22,23^, although previous data suggested that mitochondrial SHMT2 is mainly working to catabolize serine into glycine, while SHMT1 mainly produce serine from glycine in the cytosol^24^. To assess the effect of SHMTs knockdown on intracellular serine levels, we determined the amount of this amino acid by gas-MS; as shown in Fig. 3A, upon knocking down SHMT1, the intracellular serine decreases, suggesting that SHMT2 is unable to sustain serine production, also when high levels of extracellular glycine and formate are available (400 and 500 μM, respectively). On the contrary, SHMT2 knockdown has no effect on serine levels (Fig. 3A), suggesting that the SHMT1 isoform plays a pivotal role in controlling cytosolic serine levels.

We then assayed if the migratory ability of A549 and H1299 cells is affected by SHMTs knockdown, in the presence of glycine or serine. As shown in Fig. 3B, in the presence of high levels of glycine, SHMT1 knockdown dramatically reduces migration, while SHMT2 knockdown does not significantly alter cell behavior, again suggesting that the cytoplasmic glycine-to-serine conversion plays a key role in this context. On the other hand, the knockdown of SHMT1 slightly increases the migratory ability of A549 and H1299 cells in the presence of high levels of serine, an effect possibly due to the lack of SHMT1-mediated serine-to-glycine conversion which results in serine accumulation in the cytosol (Fig. 3C). SHMT1 is known to be able to catalyze the serine-to-glycine conversion in presence of increased levels of serine. An increase in migration is also seen after SHMT2 knockdown, suggesting that the accumulation of cytoplasmic serine (which is not consumed in the mitochondria under these conditions) controls the migratory process (Fig. 3C). These results are summarized in the scheme in Fig. 3D.

We validated these data in the presence of FBS as chemoattractant. The results shown in Fig. 3E demonstrate that the knockdown of SHMT1 reduced the migratory ability of LUAD cells also under these conditions, while knockdown of SHMT2 induced an increase in migratory process. Therefore, cytoplasmic serine starvation induced by SHMT1 knockdown limits the migratory ability of the cancer cells also in the presence of a chemoattractant.

### Shmt1 and shmt2 expression increases in patients during lung cancer progression

**Fig. 4:**
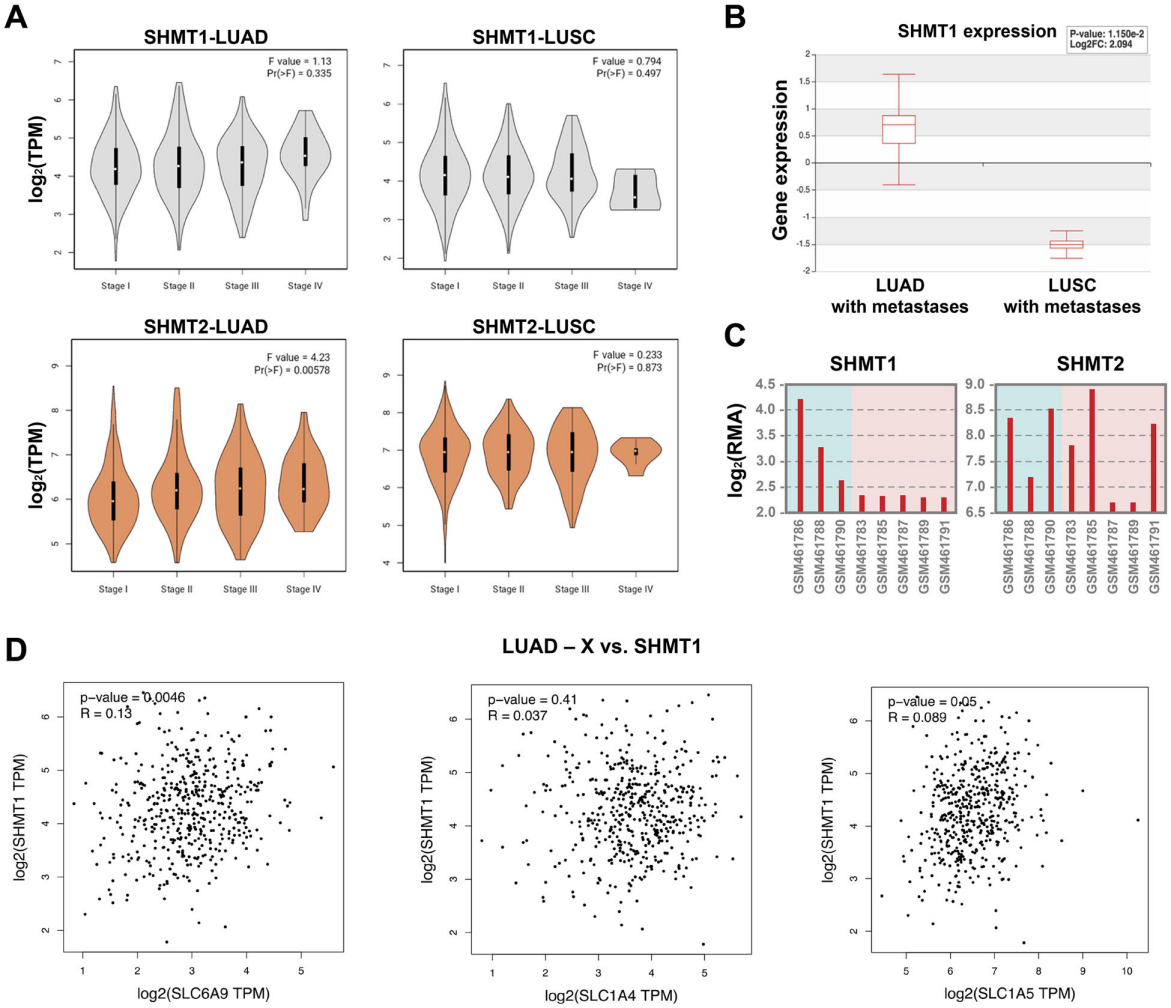
Shmt1 and shmt2 expression in patients during lung cancer progression. **A** Analysis of shmt1 and shmt2 related to patient pathological stage represented with the violin plots, Log2 (TPM + 1) for log scale. **B** shmt1 and shmt2 gene expression in lung adenocarcinoma with metastasis vs. lung squamous cell carcinoma with metastasis. **C** shmt1 and shmt2 gene expression in metastatic sites of different tumors from GSE18549 series^45^. Data are expressed as log2 RMA signal intensity. GSM461786, GSM461788, GSM461790, lung adenocarcinoma (primary site) to brain (metastatic site); GSM461783, breast carcinoma to brain; GSM461785, colon adenocarcinoma to brain; GSM461787, esophageal adenocarcinoma to brain; GSM461789, colorectal adenocarcinoma to brain; GSM461791, breast mucinous adenocarcinoma to brain. **D** Pearson correlation analysis of SHMT1 as log2(TPM) and of SLC6A9, SLC1A4 and SLC1A5 in Lung adenocarcinoma (LUAD). *P* value cutoff = 0.001. Data from TGCA and GTEx. One-way ANOVA and Student’s *t* test were used for statistical analysis (ns = not significant; *P < 0.05; **P < 0.01; ***P < 0.005).

In LUAD cancer cell lines, SHMT1 plays a major role in controlling cytosolic serine levels, which, in turn, affect cell migration, a property that is required to form metastasis. To analyze whether the metastatic potential of lung tumors is connected to the expression of SHMTs, we analyzed the expression profile of shmt1 and shmt2 in LUAD and lung squamous cells carcinoma (LUSC) patient samples, stratified according to patients pathological stage, based on TCGA clinical annotation (Fig. 4A)^25,26^. A statistically significant correlation (*P* value < 0.05) is found regarding shmt2 expression in LUAD, which is not surprising since the role of SHMT2 in supporting cell proliferation in cancer is well recognized^27^. However, by comparing only stage I with stage IV states with a two-tailed *t*-test analysis, an increased expression of shmt1 in LUAD (and a decreased expression in LUSC) can be observed (*P* value = 0.0052) (Fig. 4B). These data are in agreement with the analysis of GSE29827 data set (LUAD with metastasis vs. LUSC with metastasis), showing that shmt1 is highly upregulated in metastatic LUAD only (Fig. 4B), in agreement with our working hypothesis that the cytosolic isoform of SHMT may play an essential and unique role in the metastatic potential of this type of tumor. This trend is confirmed when comparing the expression levels of shmt1 in LUAD with respect to other primary tumors known to form metastasis in brain (Fig. 4C). We also observed a significant correlation between the expression of shmt1 and that of the glycine (SLC6A9) and serine (SLC1A5) transporters inhibited in the present study (Fig. 4D).

### Cytosolic serine levels controlled by SHMT1 modulate reactive oxygen species (ROS) formation and ATP production

**Fig. 5:**
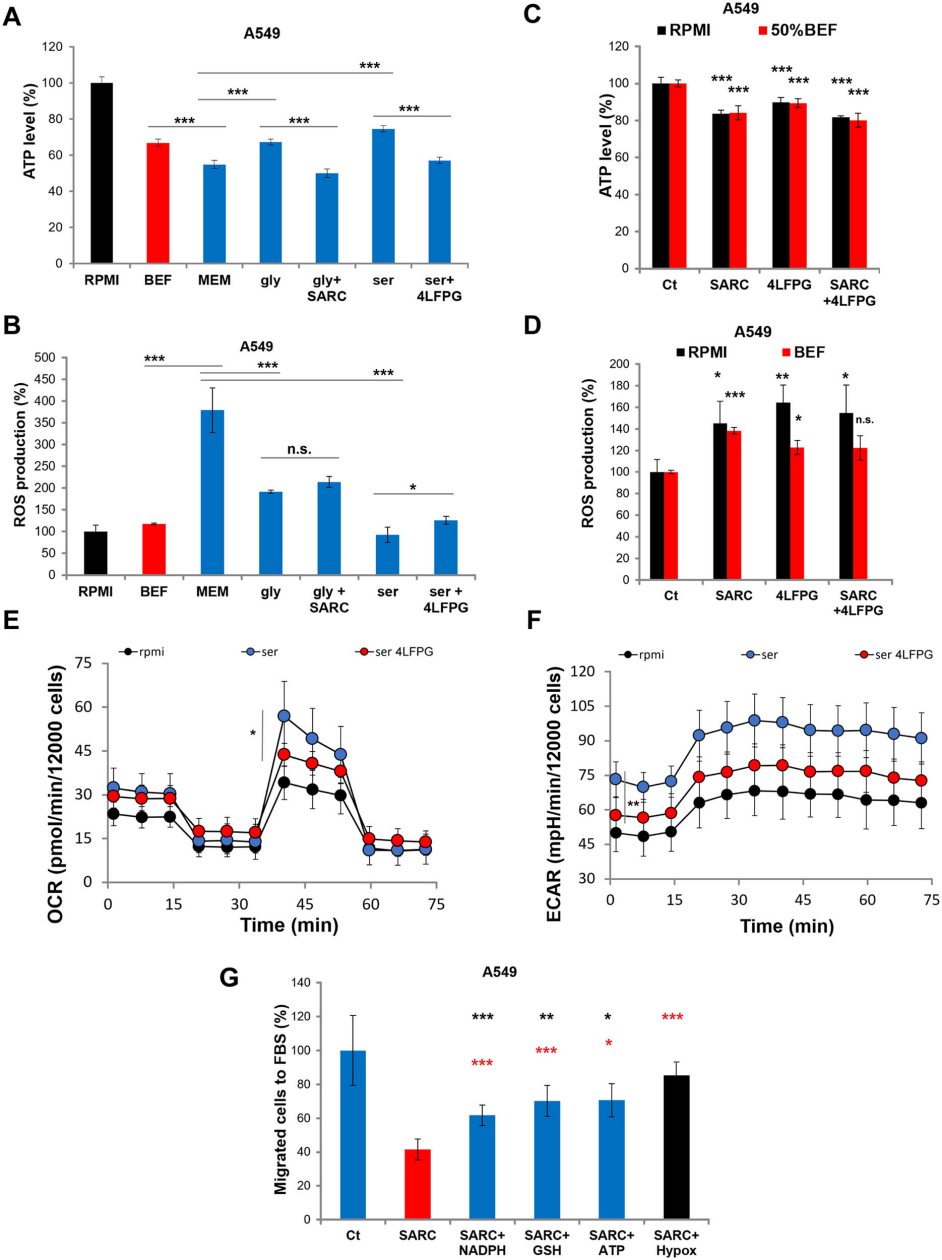
Cytosolic serine levels controlled by SHMT1 modulate ROS formation and ATP/energy profile. Effect of nutrients on ATP and ROS levels in A549 cells. The effect of 400 μM Ser ± 4LFPG or Gly ± SARC supplementations, on ATP levels (**A**) and ROS production (**B**) in A549 cells grown in MEM as compared to cells grown in complete medium RPMI or 50% brain extracellular fluid (BEF). Effect of the inhibition of Ser/Gly uptake on ATP (**C**) and ROS levels (**D**) in A549 cells grown in RPMI complete (10% FBS) or 50% BEF in MEM. The levels of the indicated parameters were assayed after incubation of the cells with 100 μM of 4LFPG and/or SARC. ATP concentration measured for RPMI (**A**) and Ct (**C**) are 29.4 ± 3.4 nmol/mg protein and 19.6 ± 1.9 nmol/mg protein, respectively. For all the experiments in (**A**–**D**) *n* = 4. OCR (**E**) and ECAR (**F**) analysis (*n* = 4) of A549 cells in RPMI (black) or RPMI with increased serine concentration (385 μM, blue in the figure) and in combination with 4LFPG (red); a representative experiment is shown (data are the mean of at least five wells ± SD). Asterisks refer to *P* values for serine vs. 4LFPG. *P* values for RPMI vs. serine samples are <0.01 for both OCR and ECAR (not shown). **G** Migration of A549 cells to 50% serum after treatment with 25 μM SARC alone or in the presence of 50 μM NADPH or GSH or with 25 μM ATP or hypoxanthine^12,46^. The graphs represent three independent experimental replicates. *P < 0.05; **P < 0.01; ***P < 0.005.

Cancer cell migration is a very complex process, which generally involves changes in cell shape driven by the cytoskeleton and production and utilization of energy derived from ATP. To assess whether ATP levels are affected in lung cancer cell lines when serine intracellular level decreases, we used minimal medium (MEM) with or without serine or glycine + formate supplementation. A significant decrease in ATP levels and a parallel increase in ROS formation are observed when growing lung cancer cells in MEM, with respect to RPMI (Fig. 5A, B). Serine or glycine supplementation reverts this effect, by increasing ATP production of 37% and 24% (Fig. 5A) and decreasing ROS production of 76% and 50%, respectively (Fig. 5B). Interestingly, supplementation with BEF also significantly increases ATP levels (about 22%) and reduces ROS production (about 69%) with respect to MEM (Fig. 5A, B). Using the inhibitors of glycine or serine uptake, the rescue seen in the presence of serine/glycine and BEF is significantly but not fully impaired (Fig. 5A–D). This partial inhibition can be explained by the limited efficacy of these inhibitors on the relative transporter (see Supplementary Fig. 1A). Overall these data underlie that these metabolites are responsible for the modulation of energy metabolism of lung cancer cells under these conditions.

To better clarify the link between cytoplasmic serine levels and cellular ATP, we studied mitochondrial respiration, by evaluating the rate of oxygen consumption rate (OCR), and glycolysis, by measuring the extracellular acidification rate (ECAR). The OCR and ECAR were measured on A549 cells growth in RPMI or RPMI enriched in serine at the concentration of maximal migratory ability shown in Fig. 1C (385 μM). Addition of serine in the growth medium significantly increases both respiration and glycolysis (Fig. 5E, F); the combined treatment with serine and the inhibitor 4LFPG significantly reduces the observed effect on ECAR, suggesting that cytoplasmic serine mainly modulates ATP production by acting on glycolysis (Fig. 5F). The influence on mitochondrial respiration is unmasked upon stressor treatment, being the maximal respiratory capacity of 4LFPG-treated significantly reduced as compared to the control experiment (Fig. 5E).

To further validate the hypothesis that the cytosolic serine-dependent modulation of ATP and ROS levels affects the migratory ability of A549 cells, we performed rescue experiments in the presence of extracellular ATP (known to be internalized by LUAD cells^28^), antioxidants molecules (GSH and NADPH), and hypoxanthine. The latter metabolite affects several pathways crucial for energy metabolism, including those leading to purine and ATP production, acting as a checkpoint reservoir of purine bases in the salvage pathway and is involved in cytoskeleton dynamics^29^. As shown in Fig. 5G, with respect to the reduced cellular motility observed in presence of SARC, the supplementation with ATP, GSH, or NADPH partially rescues the migratory phenomenon, while hypoxanthine administration is able to fully recover cell motility, further indicating that both ATP increase and cytoskeleton remodeling are required to ensure cell motility^29^. Altogether, these data indicate that a drop in the cytoplasmic levels of serine results in a significantly reduced ATP production and an increased ROS production, which impairs cell motility.

### AMP kinase is the sensor of serine starvation modulating cell migration

The data reported above suggest that pulmonary adenocarcinoma cells, also in the presence of a modest reduction of the cytoplasmic serine, activate a defensive mechanism that shuts down some cellular functions deemed superfluous, such as the migratory capacity, possibly to exploit the residual serine in other reactions considered essential, such as those supporting proliferation, which is indeed unaltered in the presence of the inhibitors of serine/glycine uptake.

**Fig. 6:**
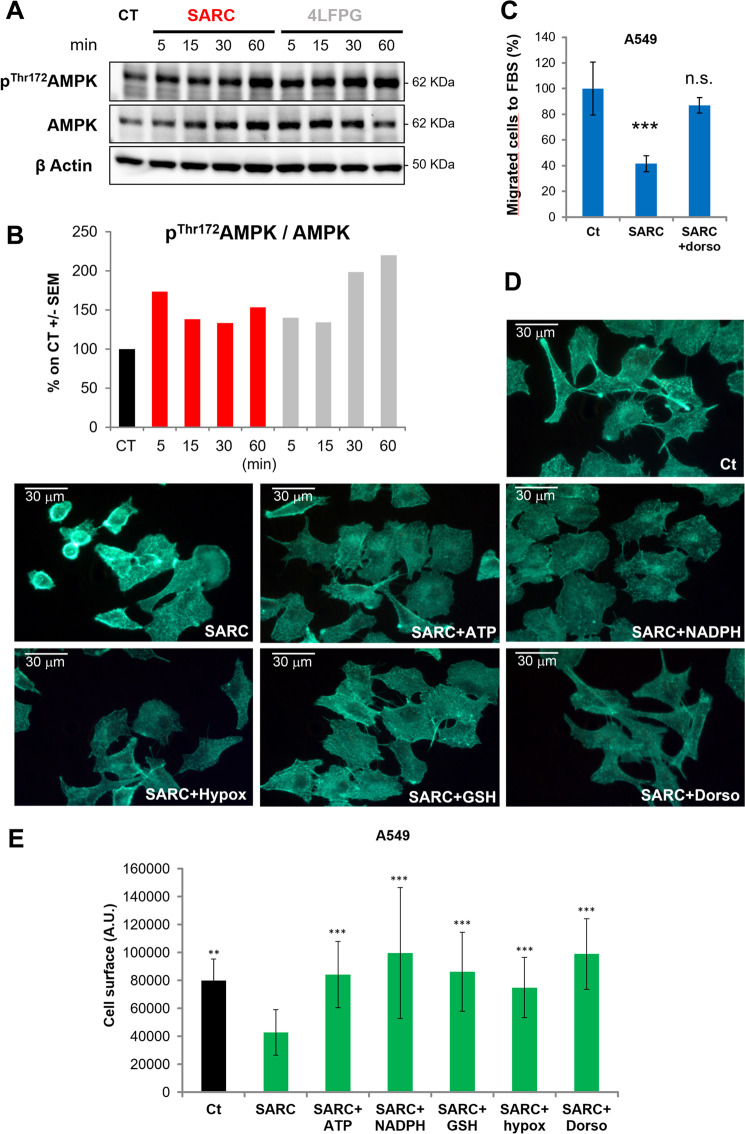
AMP kinase is the sensor of serine starvation modulating cell migration. **A** Western blot images showing a time course of p-AMPK^Thr172^ and total AMPK protein levels in presence of 100 μM SARC or 4LFPG; actin was used as loading control. **B** Densitometric evaluation of the p-AMPK^Thr172^/AMPK ratio. Data are expressed as percentage of CT set as 100%, the experiment has been repeated three times with similar results. **C** Migratory ability of A549 cells to serum in the presence of sarcosine and dorsomorphin. Cells pretreated for 3 h with 25 μM SARC ± 2 μM dorsomorphin (dorso) and non-pretreated control cells were tested in Boyden chamber assay (Ct) (*n* = 3). **D** F-actin visualization using phalloidin in A549 cells, after incubation for 24 h with each of the indicated compounds (25 μM SARC plus 25 μM ATP or hypoxanthine (hypox), 200 μM GSH, 150 μM NADPH, or 0.5 μM dorso). **E** Cell surface analysis of three images taken at the end of the experiment shown in (**D**); average ± SD is shown. **P < 0.01; ***P < 0.005.

One of the main modulators able to sense the increase in ROS and the decrease in ATP and to modulate cellular activities is the kinase activated by AMP (AMPK)^30^. To test the possible involvement of AMPK in the inhibition of cell migration obtained by using SARC or 4LFPG, we evaluated AMPK activation in the presence or absence of these inhibitors by measuring the phosphorylation level of AMPK Thr172 per total protein levels ratio (p^Thr172^AMPK/AMPK) by western blot analysis. Both drugs increase the phosphorylated form of the protein starting from 5 to 15 min of the treatment indicating activation of the enzyme (Fig. 6A, B). To further demonstrate the direct involvement of AMPK in the SARC/4LFPG-mediated inhibition of cell motility, we used dorsomorphin, a known specific inhibitor of AMPK. When dorsomorphin is used at the concentration of 2 μM, it completely reverts the inhibitory effect induced by SARC demonstrating a direct involvement of AMPK in the mechanism controlling the migration of A549 cells (Fig. 6C). In the absence of SARC, migration is unaffected by AMPK inhibition by dorsomorphin (Supplementary Fig. 3)^31^.

One of the main functions of AMPK is to inhibit cell migration, even if the molecular details are still poorly understood. AMPK is able to attenuate the production of the subcellular structures responsible for exploration of the microenvironment (filopodia) and for the movement (lamellipodia)^32^. We therefore used phalloidin, a molecule capable of specifically binding to filamentous actin to further study the connection between cytoplasmic serine starvation and filopodia/lamellipodia formation. As shown in Fig. 6D, the A549 cells show the characteristic structures of lamellipodia and filopodia, which are severely affected when the cells are treated with SARC. As expected, evaluation of cell surface indicates a reduction in this parameter when the cells are treated with SARC (Fig. 6E). Co-treatment of the cells with SARC together with ATP, NADPH, GSH, hypoxanthine, or dorsomorphin increases the production of lamellipodia and filopodia (Fig. 6D), and results in a parallel increase in the cell surface (Fig. 6E), validating our hypothesis that podia formation is inhibited by ROS upregulation and ATP reduction in a process mediated by AMPK.

## Discussion

Tumors adapt to the nutrients present in the microenvironment by rewiring their metabolism in order to optimize cell growth and eventually escape from primary sites when the supply of nutrients or oxygen is low and invade novel and richer environments by forming metastasis. In particular, LUAD frequently form brain metastases, often related to poor prognosis in advanced stages patients. We showed here that the metabolism of specific amino acids, i.e., serine and glycine, found in the brain microenvironment (represented here by BEF), plays an active role in the control of adenocarcinoma cell motility. The uptake of extracellular serine and glycine is required for migration, and motility of cancer cells can be effectively impaired by using inhibitors of the serine/glycine transporters SLC1A4/SLC1A5 and GlyT1, which are involved in this process. Furthermore, the data presented here prove that maintenance of elevated cytosolic serine levels by the OCM cytosolic enzyme SHMT1 is a key event in the control of adenocarcinoma cell motility, since the lack of SHMT1 heavily affects this property, while SHMT2 knockdown has no effect. On the other hand, SHMT2, the mitochondrial isozyme, overexpressed in many tumors^27^, is required for cancer cell proliferation since it provides the one carbon units essential to build biosynthetic precursors. In agreement with our results, the analysis of cancer patients’ samples show that, as expected, shmt2 expression gradually increases during the various stages of disease progression, while shmt1 expression increases only in the more advanced stages; moreover, shmt1 expression nicely correlates with that of the transporters involved in this work. These observations argue for a more direct involvement of SHMT1 compared to SHMT2 in the processes of metastasis formation. Furthermore, shmt1 expression was found to be significantly elevated in metastatic LUAD compared to LUSC; interestingly, within the subtypes of non-small cell lung cancer, brain metastases are more common with LUAD than with LUSC^33^.

Our results unveil a novel link between cytosolic serine levels, energy metabolism, and control of cell motility. We show that serine is required to maintain ATP levels and avoid ROS formation; interfering with its uptake or bioavailability reshapes the energy metabolism promoting a small but significant drop in ATP levels, mainly due to a reduction of glycolysis, which is sufficient to completely block cell migration. While the metabolic effects of serine starvation on ATP and ROS levels were previously observed in other cancer cells^12^, the effect on cell motility was yet unknown. A sufficient level of ATP is required to support cell motility: if ATP drops and ROS increase over a threshold because serine is not available, the intracellular sensor AMPK is activated, and cytoskeleton dynamics is impaired^34,35^. Therefore, even the limited effect on the intracellular levels of ATP and ROS induced by SARC or 4LFPG is sufficient to induce a complete arrest of cell migration.

**Fig. 7:**
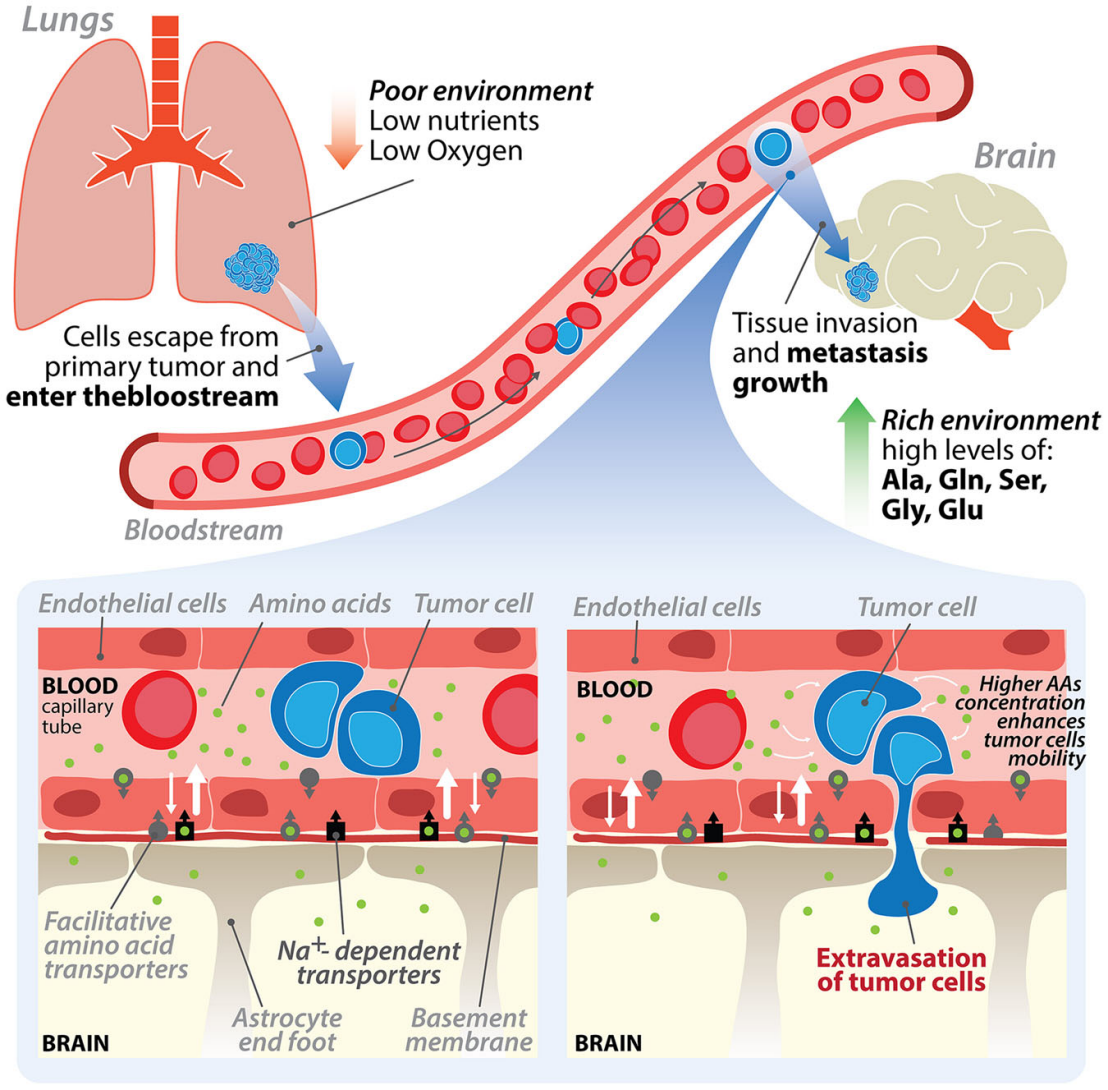
Brain-to-blood efflux model. The scheme depicts the hypothesis that cancer cell migration from the primary tumor site and invasion of the brain parenchyma is prompted by the high levels of amino acids, including serine and glycine, whose effect on cell motility was shown in this work.

In summary, serine and glycine levels in the tumor microenvironment were found to control cell motility acting on ATP and ROS intracellular levels via AMPK. How could this observation be related to the preferential migration of LUAD cells in the brain? We suggest an original working hypothesis on how these events may favor invasion of brain by LUAD cells and formation of metastasis, which is depicted in Fig. 7. We hypothesize that our experiments mimic the situation occurring at the vessel/brain–blood–barrier (BBB) interface; at this level, cancer cells are able to form clots, thereby slowing down the blood flux^36^. In this peculiar microenvironment, high levels of neurotransmitters including serine and glycine are released into the blood through the process called brain-to-blood efflux. Underlying this physiological phenomenon is the brain’s need to reduce the level of neurotransmitters that remains in the extracellular fluid at the end of a stimulus, to avoid further unwanted stimulations. The released neurotransmitters are immediately reabsorbed, in part by the nerve terminal and by the adjacent glial cells and in part by endothelial cells of the BBB in the surrounding area via specific transporters present only on the abluminal side of these cells (on the brain side) and released into the lumen of the micro vessels^37^. We envisaged that, as a consequence of the increase of local serine/glycine concentration, the migratory ability of cancer cells is enhanced, prompting the cells to move into the target organ (Fig. 7). We believe that this hypothesis frames in a consistent molecular picture and provide a logic basis to explain the high propension of LUAD cells to invade the brain. Our observation identifies a metabolic signature that might be employed as a prognostic factor of tumors, which have a high probability to form metastasis in the brain microenvironment and/or could identify metabolic dependencies to target even in brain-resident tumors. Inhibition of these processes is likely an effective strategy to diminish lung cancer brain metastasis from primary tumors, an unmet medical need.

## Materials and methods

### Cell lines

A549, H1299 and H460 cells were purchased from ATCC (Manassas, VA, USA). The cells were grown in RPMI-1640 medium (Corning) or in Minimum Essential Medium Eagle MEM (Sigma-Aldrich) as previously described (Marani et al.^38^) supplemented with 0.4 mM of each indicated metabolite (Sigma-Aldrich) or 50% BEF.

### Cell migration assay

Migration test was performed using Boyden chambers (NeuroProbe, Gaithersburg, MD USA). Briefly, 50 × 10^3^ cells were suspended in RPMI serum free (SF) and then plated in the upper compartment of the Boyden chamber. The two chambers were separated by a 13-mm-diameter polyvinylpyrrolidone filter with 8.0 μm pore size (Whatman International, Maidstone, UK). For the chemoattraction bioassay, BEF or RPMI-SF supplemented with 0.4 mM of Ser or Gly or 50% FBS were placed in the lower chamber (Fig. 1A). For the cell motility capacity test, the cells in the upper compartment were resuspended in RPMI-SF supplemented with increasing concentrations of Ser or Gly with/without a 25 µM 4LFPG or SARC. The lower chamber contains only RPMI-SF (Fig. 1D). For both assays, the chambers were then incubated for 6 h at 37 °C under 5% CO_2_. The filters were recovered and fixed with ethanol 70%. Non-migrating cells were removed from the upper surface of the filter using a cotton swab, and migrating cells were stained with 0.1% crystal violet and quantified by counting them using an optical microscope. Each experiment was done at least in triplicate and the stained cells were counted as the mean number of cells per six random fields for each replicate.

### Western blot analysis

Cells were harvested, total protein extracts were prepared in RIPA buffer, and 15 μg of proteins was separated by 4–15% gradient SDS-PAGE as previously described^39^. Briefly, the proteins were then transferred onto nitrocellulose membranes and incubated over night at 4 °C with the following primary antibodies: AMPK and pThr172 AMPK^39^, and anti-SHMT1 and β-actin^21^. Subsequently, membranes were incubated at RT with the respective horseradish peroxidase-conjugated secondary antibodies and the relative abundance of each protein was calculated using Image Lab 6.0.1 software (Bio-Rad Laboratories).

### siRNA cell transfection

Cells were seeded at the density of 10^5^ cells/well on six-well culture plate in RPMI. Twenty-four hours after seeding, the cells were transfected with 15 nmol/l siRNA with Qiagen AllStars RNAi Controls scrambled sequences, or siRNA sequences against shmt1 or shmt2 (Qiagen, Hilden, Germany) using specific siRNA sequences as described previously in ref. ^21^ using the JetPRIME reagent (PolyPlus), according to the manufacturer’s instructions. The medium was replaced after 16 h by fresh medium, and the cells were used for migration test 24 h after transfection, or 48 h later for western blot analysis.

### Reactive oxygen species (ROS) quantification

Quantification of ROS production in all cell types was measured after 48 h treatment, using the fluorescent probe 2′,7′-dichlorodihydrofluorescein diacetate (Sigma-Aldrich, USA) as previously described^40^.

### Staining of cellular F-actin with phalloidin

The cells were grown on poly-lysine adhesion slides (Thermo Scientific) maintained in cell culture dish (Ø: 100 mm/height 22 mm). The cells were then simultaneously treated or not with SARC and/or GSH, NADPH, ATP, hypoxanthine, or dorsomorphin. Twenty-four hours later, the cell medium was removed, and the F-actin was stained using FITC-phalloidin (1:1000, Santa Cruz Biotechnology) according to the manufacturer’s instructions. Coverslips were applied to each slide and cell actin filament polymerization was visualized using fluorescence microscope Zeiss AXio (×400 magnification) (Carl Zeiss, Oberkochen, Germany). The images were collected using the Zen 2011 software (Zeiss) and were identically adjusted for background using Photoshop Cs6 (Adobe System, San Josè, CA, USA).

### Cell surface analysis

ImageJ (US National Institutes of Health, Bethesda, Maryland, USA) macros “Measure Cell Surfaces” tool (http://dev.mri.cnrs.fr/projects/imagej-macros/wiki/Measure_Cell_Surfaces) were used to perform cell surface analysis of pictures taken after the experiments shown in Fig. 6D following the instructions.

### Total cellular ATP content assay

The ATP level was measured in cell lysates as previously reported^41^. Briefly, cells were detached with trypsin, washed once with HBSS, and resuspended in a buffer containing 0.25 M sucrose, 50 mM HEPES, 0.5 mM EDTA, 4 mM MgSO4, pH 7.4. Aliquots of cells were lysed in DMSO and total cellular ATP was measured using a Luciferin–Luciferase assay kit (ATP bioluminescent assay kit, Merck KGaA, Darmstadt, Germany) according to the manufacturer’s protocol. The amount of ATP measured was normalized to the protein content determined by a modified protocol of the Lowry method as reported^42^.

### Brain extracellular fluid (BEF) preparation

Mice B6EiC3SnF1/J were housed in clear Plexiglas cages (20 × 22 × 20 cm) under standard conditions with a T° of 22 ± 2 °C and 70% humidity, a 12 h light/dark cycle and free access to food and water. All procedures were performed in strict compliance with the Italian National Laws and the European Communities Council Directives. Collection of BEF: 3-month-old mice were anesthetized (Xylazine 10 mg/kg IP) and intracardially perfused with ice-cold PBS, then we used a protocol commonly used to prepare primary cell cultures from mouse brain^43^. Brains were rapidly removed and placed into ice-cold HBSS; the hemispheres were cut into small pieces and gently disrupted in a glass–teflon homogenizer then passed through a 100 μm nylon cell strainer (Becton-Dickinson). Suspension was centrifuged (800 × *g*, 10 min, RT) and the liquid part collected.

### Gas chromatography-mass spectrometry

All chemicals and solvents (Carlo Erba, Italy) were of analytical grade. N-tert-butyldimethylsilyl-N-methyl-trifluoroacetamide and 3,4-dimethoxybenzoic acid (C9H10O4) were purchased from Sigma-Aldrich (Germany). Ser and Gly were analyzed according to the method of Molnár-Perl et al.^44^ with slight modification. Briefly, cell pellets were resuspended in 0.1 ml of 0.1 M HCl/acetonitrile (1:1 vol/vol) solution and prepared and subjected to GC-MS analysis. GC-MS analyses were performed with an Agilent 6850A GC coupled to a 5973N quadrupole mass selective detector (Agilent Technologies, Palo Alto, CA, USA). Chromatographic separations were carried out with an Agilent HP5ms fused-silica capillary column (30 m × 0.25 mm i.d.) coated with 5%-phenyl-95%-dimethylpolysiloxane (0.25 μm) as stationary phase. Injection mode: spitless at 250 °C. Column temperature program: 80 °C (1 min) ramped to 300 °C at a rate of 20 °C/min, and held for 15 min. The carrier gas was helium at a constant flow of 1.0 ml/min. The spectra were obtained in the electron impact mode at 70 eV ionization energy; ion source 280 °C; ion source vacuum 10–5 Torr. MS analysis was performed in TIC (mass range scan from *m/z* 50 to 600 at a rate of 0.42 scans s −1) and SIM mode. GC-SIM-MS analysis was performed selecting the following ions: *m/z* 218 for Gly, *m/z* 288 for Ser, and *m/z* 239 for C9H10O4.

### Seahorse XF analyzer respiratory assay

Cellular OCR and ECAR were detected using XF Cell Mito Stress Test (Agilent) measured by the extracellular flux analyzer XFe96 (Seahorse Bioscience, Houston, TX, USA) as previously reported^43^. A549 cells were cultured on XFe culture miniplates (12,000/well). Cells have been cultured with serine 385 μM and or 4LFPG 100 μM for 24 h before the analysis. Two independent experiments were carried out. The sensor cartridge for XFe analyzer was hydrated in a 37 °C non-CO_2_incubator a day before the experiment. According to the manufacturer instructions, stressors concentrations were optimized and added as follows: 1 μM oligomycin as complex V inhibitor, 0.5 μM FCCP (uncoupler agent), and 0.5 μM rotenone/antimycin A (inhibitors of complexes I and III).

### Statistical analysis

All the data are the mean ± standard deviation of at least three independent biological experiments. Paired samples data were analyzed with Student’s *t* test; all the others statistical analysis were performed using one-way ANOVA followed by the Bonferroni post hoc comparison test. *P* < 0.05 was considered significant.

## Supplementary information

Supplementary information
