# Supplementary material for: Cytosolic serine hydroxymethyltransferase controls lung adenocarcinoma cells migratory ability by modulating AMP kinase activity
Source: Cell Death Dis. 2020 Nov 26;11(11):1012. doi: 10.1038/s41419-020-03215-0 (PMC7691363; doi:10.1038/s41419-020-03215-0)
Supplement: Supplementary file 1 — Supplementary information [file 41419_2020_3215_MOESM1_ESM.docx]

Supplemental information for

**Cytosolic Serine Hydroxymethyltransferase controls lung adenocarcinoma cells migratory ability by modulating AMP Kinase activity**

**Authors**

Amani Bouzidi^a^, Maria Chiara Magnifico^a,1^, Alessandro Paiardini^a^, Alberto Macone^a^, Giovanna Boumis^a^, Giorgio Giardina^a^, Serena Rinaldo^a^, Francesca Romana Liberati^a^, Clotilde Lauro^b^, Cristina Limatola^b^, Chiara Lanzillotta^a^, Antonella Tramutola^a^, Marzia Perluigi^a^, Gianluca Sgarbi^c^, Giancarlo Solaini^c^, Alessandra Baracca^c^, Alessio Paone^a,2,*^ and Francesca Cutruzzolà^a,2,*^.

**Affiliations**

^a^ Department of Biochemical Sciences A. Rossi Fanelli, Laboratory Affiliated to Istituto Pasteur Italia, and ^b^ Department of Physiology and Pharmacology V. Erspamer, Sapienza University of Rome, Piazzale A. Moro 5, 00185 Rome, Italy

^c^ Department of Biomedical and Neuromotor Sciences, University of Bologna, Via Irnerio 48, 40126 Bologna, Italy

**^1^Present Address:** Department of Biosciences, Biotechnologies and Biopharmaceutics, University of Bari “Aldo Moro”, Via Orabona 4, 70121 Bari, Italy

**^2^Corresponding author:** To whom correspondence may be addressed at [francesca.cutruzzola@uniroma1.it](mailto:francesca.cutruzzola@uniroma1.it) or [alessio.paone@uniroma1.it](mailto:alessio.paone@uniroma1.it).

**^*^Equal contribution:** Alessio Paone and Francesca Cutruzzolà contributed equally to this work as last authors.

**This file contains:**

**Supplementary Fig. S1-S3**

**Supplementary Table S1**


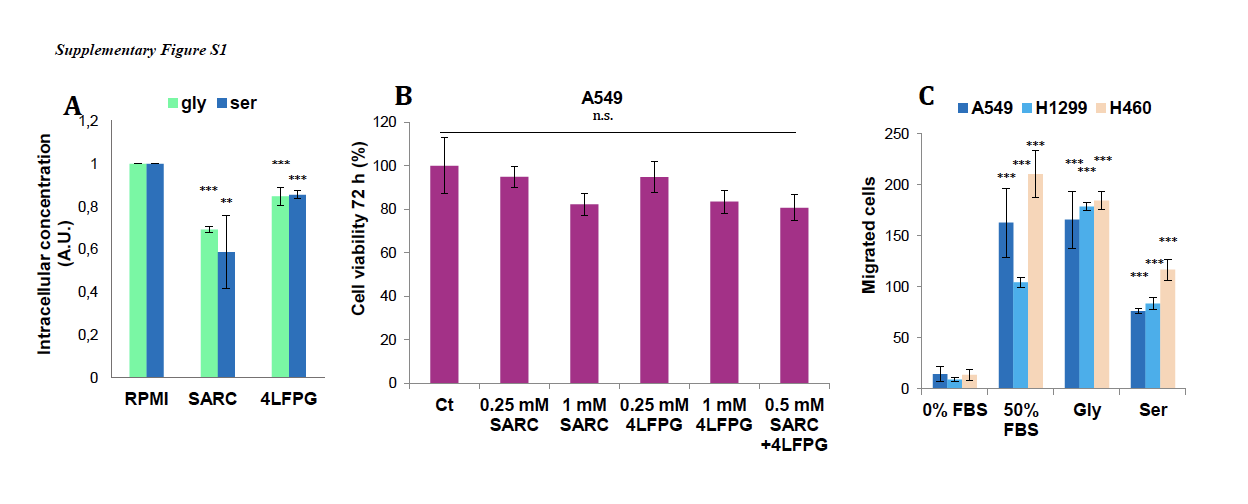


**Supplemental Figure 1**

(A) Intracellular Ser and Gly concentration after 48hrs incubation of A549 cells with 150 μM sarcosine or 4LFPG (n=3). (B) A549 cells proliferation after 72h incubation with 0, 0.25 and 1mM SARC or 4LFPG or with 0.5mM SARC plus 4LFPG (n=4). (C) Migrated cells to 0% FBS, 50% FBS, 400 μM Ser or 400 μM Gly observed for three different lung adenocarcinoma cell lines: A549, H1299, H460 (n=4). One-way Anova was used for statistics (ns: not significant; **: p< 0.01; ***: p <0.005).


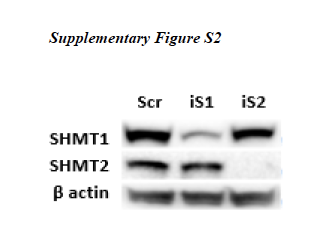


**Supplemental Figure 2**

Validation of the SHMTs knockdown experiments. Western blot on A549 cells interfered for either SHMT1 (iS1) or SHMT2 (iS2). Bands of SHMT1 and SHMT2 proteins were normalized against that of B-actin. The Experiment has been repeated 3 times with similar results.


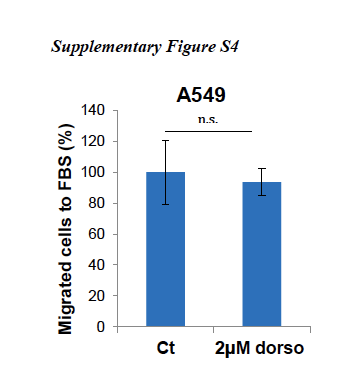


**Supplemental Figure 3**

Effect of 3 h treatment with dorsomorphin on A549 cell migration (n=3).

| Metabolite in BEF | Concentration ± SD (A.U.) |
| --- | --- |
| Lactate | 1,76875 ± 0,360 |
| Isoleucine | 0,50025 ± 0,105 |
| Glutamate | 0,46 ± 0,095 |
| Aspartate | 0,3765 ± 0,102 |
| Alanine | 0,316 ± 0,143 |
| Valine | 0,259 ± 0,063 |
| Glycine | 0,09725 ± 0,036 |
| Serine | 0,0925 ± 0,050 |
| Leucine | 0,06225 ± 0,019 |
| Phenylalanine | 0,05225 ± 0,007 |
| Threonine | 0,04475 ± 0,015 |
| Methionine | 0,00975 ± 0,005 |

**Supplemental Table 1**

GC-MS analysis showing the levels of different metabolites found in BEF. The data are obtained by averaging 4 independent experiments.
